# Support for alcohol policies from drinkers in the City of Tshwane, South Africa: Data from the International Alcohol Control study

**DOI:** 10.1111/dar.12554

**Published:** 2017-05-10

**Authors:** Charles D. H. Parry, Pamela Trangenstein, Carl Lombard, David H. Jernigan, Neo K. Morojele

**Affiliations:** ^1^ Alcohol, Tobacco and Other Drug Research Unit South African Medical Research Council Cape Town South Africa; ^2^ Department of Psychiatry Stellenbosch University Cape Town South Africa; ^3^ Department of Health, Behavior and Society Johns Hopkins Bloomberg School of Public Health Baltimore USA; ^4^ Biostatistics Unit South African Medical Research Council Cape Town South Africa; ^5^ Alcohol, Tobacco and Other Drug Research Unit South African Medical Research Council Pretoria South Africa; ^6^ School of Public Health University of the Witwatersrand Johannesburg South Africa; ^7^ School of Public Health and Family Medicine University of Cape Town Cape Town South Africa

**Keywords:** alcohol, policy, South Africa, heavy drinker

## Abstract

**Introduction and Aims.** South Africa is considering a range of alcohol policy reforms. This study aims to determine the magnitude of public support for 13 alcohol policies in the Tshwane Metropolitan Municipality and whether this varies by demographic factors and heavy drinking status. **Design and Methods.** Data are from the South African arm of the International Alcohol Control study, a household survey of adult drinkers using a multistage stratified cluster random sampling design. The sample included 1920 drinkers aged 18–65 years (62% men), with complete drinking data for 16 drinking locations on 955 persons (510 heavy and 445 not heavy drinkers). **Results.** Over half (53%) of the sample were found to be heavy drinkers. Support varied by alcohol policy, ranging from 31% to 77%, with support above 50% for 11 of the 13 policies. Policy support was higher for policies increasing the purchase age to 21 years (77%), addressing drink driving (58–76%) and restricting physical availability (60–66%). There was slightly less support for policies restricting alcohol marketing (59%) or for policies increasing the price of alcohol (34–58%), especially if no justification was given or the funds were not earmarked. Policy support differed by age, gender, heavy drinking status and income. **Discussion and Conclusions.** Public support from adult drinkers for a range of alcohol policies is extensive and, as found elsewhere, was strongest for raising the minimum drinking age and lowest for increasing prices. The support from drinkers to increasing controls on alcohol could be one lever to getting control measures implemented. [Parry CDH, Trangenstein P, Lombard C, Jernigan DH, Morojele NK. Support for alcohol policies from drinkers in the City of Tshwane, South Africa: Data from the International Alcohol Control study. *Drug Alcohol Rev* 2017;00:000‐000]

## Introduction

Excessive alcohol use is an important driver of poor health in South Africa 1. In 2010, adult drinkers drank on average 27.1 L of pure alcohol, the highest in Africa 2. However, 59.4% adults abstained, suggesting that the minority of drinkers drink heavily 2. This drinking pattern incurs substantial alcohol‐related harms, and in 2015, alcohol was identified as the fifth leading risk factor for death and disability in this country 3. Factors facilitating the harmful use of alcohol in South Africa include the low price of alcohol, the density of outlets in urban areas, the poor regulation of sales as over half the on‐consumption and off‐consumption sale of alcohol occurs in unlicensed outlets and the lack of government oversight of marketing 4.

In 2011, the South African government hosted an anti‐substance abuse summit that stimulated various alcohol policy reforms for consideration: improving the regulation of hours/days of alcohol sale and increasing the minimum drinking/purchase age from 18 to 21 years 5; reducing the allowable blood alcohol concentration for drivers from 0.05% to 0.02% 6; designating malt and sorghum beer as ‘liquor’ 7; and banning alcohol advertising except at points of sale 8.

Various factors contribute to the selection and implementation of public policies, including evidence of effectiveness, pressures from alcohol producers and retailers, the desire to generate revenues and the cost of implementing the policies 9, 10, 11. It is generally recognised that public opinion has an influence on policy, but equally that policies can influence public opinion 10, 12, 13, 14. Public attitudes can constrain the policies supported by governments and this might explain why more effective interventions (e.g. restricting physical and economic availability) are less likely to be implemented and why popular but ineffective policies (e.g. school‐based education programs) are more likely to be implemented 9, 12, 15, 16. The impact of public opinion on alcohol policies has been found to change over time 16, 17 and to be influenced by factors such as concerns about the greater availability of alcohol, increased understanding by the public of the harm caused by others' alcohol use and the intrusiveness of the policy 9, 16, 18.

Across a variety of developed countries, support for more restrictive alcohol policies tends to be highest among women 9, 11, 15, 16, 18, 19, older people 9, 15, 18, 19 and light or non‐drinkers 9, 11, 15, 16, 18. Mixed findings have been observed regarding the effect of education and income 9, 15, 18. Research conducted has also found that support for alcohol policies is higher when those policies are aimed at high‐risk venues and populations 12.

Most of the public opinion research on alcohol policies is specific to the developed world 9, 10, 11, 12, 13, 14, 15, 16, 17, 18, 19, 20, 21. Apart from studies from South Korea, Puerto Rico and Brazil 22, 23, 24, little is known about public support for tightening alcohol policies in developing countries. Levels of support differ substantially across these countries, with 10 out of 12 policies proposed in South Korea receiving less than 50% support 2, whereas in Puerto Rico 23, support ranged from 67% to 95% across 20 items, and in Brazil 24, it ranged between 55% and 74% across six policy measures. These studies also found that women, older persons and persons who drank less were more likely to approve more restrictive alcohol policies 23, 24. However, extrapolating from studies conducted in a few other developing countries to the South African policy environment may be problematic because of the latter's unique drinking patterns and differing existing policy climate 2. The views of non‐drinkers regarding alcohol policy options are likely to be more supportive than those of moderate and heavy drinkers, but it is the latter whom policymakers would expect to oppose many policy measures 10. The purpose of this study was to measure public support from adult drinkers for alcohol policies in South Africa and to determine whether demographic and alcohol consumption factors are associated with such support.

## Methods

### Design and sampling

The data for this study are from the South African arm of the multicountry International Alcohol Control (IAC) study 25. This cross‐sectional study was conducted during 2014 in the City of Tshwane Metropolitan Municipality, located around the executive capital, Pretoria. The study used a multistage stratified cluster random sampling design, which involved selecting communities, that is, wards consisting of formal communities, informal communities and townships; census enumeration areas (EA) within selected communities; and then households within selected EAs. Townships refer to underdeveloped urban living areas that are often adjacent to informal communities comprising dwellings made of zinc and wood and having rudimentary infrastructure. From the selected households, we randomly selected one adult. Eligible participants had to have consumed alcohol in the past 6 months and be 18 to 65 years old. The target sample size of 2000 was determined by the IAC study 25.

### Measures

We adapted the standard (English) IAC questionnaire then translated and back‐translated it into Setswana and Afrikaans. It assessed demographic factors (e.g. age, gender and socioeconomic status), alcohol consumption and support for alcohol policies. The latter was obtained on a 5‐point Likert scale ranging from ‘strongly support’ (1) to ‘strongly oppose’ (5) with ‘don't know’ and ‘refuse’ options. Policy support questions were dichotomised. Strongly support (1) and support (2) were combined to indicate support, and neutral (3), oppose (4) and strongly oppose (5) were combined to indicate lack of support. Persons who responded ‘don't know’ or refused to answer the question were excluded for that policy.

Heavy drinking was defined as consuming at least 120 ml [eight standard drinks of 15 ml (12 g) of absolute alcohol] for men and at least 90 ml [six standard drinks of 15 ml (12 g) of absolute alcohol] for women on one occasion at any location at least monthly. Twelve grams of alcohol is the typical standard drink size used in South Africa. This definition, used by the IAC study 25, is higher than typically used in many surveys and by the World Health Organization, but reflects the growing questioning of the validity of the 4+/5+ binge or heavy drinking criterion 26. The alcohol consumption section of the questionnaire asked quantity and frequency for typical alcohol consumption at 16 locations (i.e. at your home; at someone else's home; at nightclubs; at sports clubs; at restaurants, cafés or coffee shops; theatres or movies; at workplaces; on plane trips within South Africa; in private motor vehicles; at sports events, races or boating; at outdoor public places such as beaches or parks; at shebeens; at bars, pubs or taverns; at hotels; and at special events such as festivals, music events or dance parties) over the past 6 months. From this, the quantity of absolute alcohol consumed was calculated as (number of containers) * (container size) * (percent absolute alcohol) for each of 13 beverage types (i.e. beer, low‐alcohol beer and home brew beer; stout; wine; spirits; mixed cocktails; liqueurs; shooters; sherry, port or vermouth; ciders; alcopops; and others) across the 16 locations. To determine the average consumption of absolute alcohol by location, the amount of absolute alcohol consumed was summed for all beverage types consumed at each location.

Nine hundred and sixty‐five adults did not report enough consumption data to calculate heavy drinking status (i.e. they had frequency data for a given location but no consumption data for that location or vice versa), and we excluded them from the analyses by heavy drinking. We categorised total annual personal income into low, medium and high. The low category included persons making (ZA)R30 000 or less ($AUS1 = ZAR10.4); the medium category included persons making greater than R30 000 but less than or equal to R200 000; and the high category included persons making more than R200 000. Four hundred and thirty‐three adults did not know or did not report total annual personal income. We excluded these persons from analyses that assessed policy support by income. Missing data occurred largely as a result of problems that occurred with the complicated programming of the software used in the tablets to handle the numerous skip patterns in the questionnaire 27.

Participants with missing consumption data did not differ from the sample on race/ethnicity (*F* = 2.18, *P* = 0.12) or income (*F* = 0.02, *P* = 0.97) but were more likely to be men (*F* = 4.76, *P* = 0.04), aged 20–44 years (*F* = 4.65, *P* = 0.002) and never married (*F* = 5.15, *P* = 0.001). Participants with missing personal income data did not differ from the sample on gender (*F* = 0.01, *P* = 0.92) or heavy drinking status (*F* = 0.29, *P* = 0.60) but were more likely to be 18–19 years old (*F* = 7.24, *P* = 0.001) and married (*F* = 49.11, *P* < 0.001), and they were less likely to be Black African (*F* = 10.07, *P* < 0.001).

### Procedures

After obtaining informed consent, participants were interviewed in their homes by trained interviewers. This approach was adopted because of the complexity of the questionnaire.

Interviews were administered on a tablet. The mean and median length of interviews were 34 and 25 min, respectively, and interviews ranged in length from 5 to 72 min. After the interview, participants received a resource card for alcohol‐related problems as well as a shopping or a cellular telephone recharge voucher worth R30. The study was approved by The Research Ethics Committee of the South African Medical Research Council.

### Survey design and analysis

Data were weighted to take into account the complex sampling design. At stage 1, wards were the primary sampling unit for the survey. Wards were stratified by region and majority race group, and this resulted in three strata and selected proportional to the population size (18 to 65 years) within each stratum. The population information from the 2011 census was used. Overall, 35 of 105 wards were selected. At stage 2, EAs were the second sampling unit. EAs were stratified by size into three strata based on the number of households (<100, 100 to <150 and 150+), and 396 EAs were selected. At stage 3, a fixed number of households (4, 6 and 8 adults, respectively) were selected by EA size to ensure the self‐weighting of this stage. A total of 2468 households were selected for a team visit and a single adult from the eligible participants selected (stage 4).

The sampling weights took the survey design into account: the oversampling of non‐Black participants at stage 1 and the number of eligible adults in the household at stage 4. Response rates were also calculated at the ward level, and the final weight was the product of the proportional and realisation weights. Post‐hoc stratification weighting was therefore applied to have the approximate census distribution in the sum of the weights across the 16 strata plus the total weight approximately equal to the census population of 2.9 million people of the Tshwane study area. Finite sampling correction information for each stage was set up for the survey design to improve precision. All of the wards selected were visited, and at the second stage of sampling, 331 of the selected 396 EAs were visited, resulting in a realisation percentage of 84% at this level. Within the 331 EAs surveyed, there were 2070 selected households at which 1932 interviews were completed that represents a realisation figure of 93%. Taking the product of the response rates in the three stages, the overall response rate was 78%. stata version 14.0 was used for the survey analysis 28. Data were weighted to take into account the underlying structure of the realised sample and the sample frame to ensure a random selection of respondents.

The χ^2^‐tests of association were used to detect differences in policy support by age, gender, total annual personal income and heavy drinking. The analysis used logistic regression to assess associations between predictors and each of the 13 policy options. Categorical predictors included gender (male and female), age (18–19, 20–24, 25–34, 35–44, 45–54 and 55–65 years), total annual personal income (low, medium and high) and heavy drinking status (yes and no). Participants who answered ‘don't know’ to a specific policy option were excluded from that logistic regression model. Participants with missing income and/or heavy drinking data were excluded from all logistic regressions. *P*‐values less than 0.05 were considered statistically significant.

## Results

### Sample characteristics

The sample included 1920 adults. The median age was 31 years; 62% were men; 77% had low income; and 88% were from urban areas (Table 1). The median amount of absolute alcohol consumed on a typical drinking occasion was 118.5 g (just under 10 standard drinks). Fifty‐three percent of the sample were heavy drinkers [95% confidence interval (CI): 47%, 56%]. Men were more likely to be heavy drinkers than women (*F* = 11.32, *P* < 0.01) and Black Africans were more likely to be heavy drinkers than White persons (*F* = 9.09, *P* < 0.01). However, heavy drinkers did not differ significantly from non‐heavy drinkers in terms of age, urbanicity or income. Over half (57.2%, 95% CI: 52%, 62.2%) of male drinkers were found to be heavy drinkers compared with four out of 10 female drinkers (41.2%, 95% CI: 32.9%, 50.1%). Over half of Black African (56.1%, 95% CI: 20.9%, 61.2%) and approximately a third of White participants (32.6%, 95% CI: 20.8%, 47.2%) were heavy drinkers.

**Table 1:** Characteristics of study participants by heavy drinking status

	Total (*n* = 1920)^a^ % (95% CI)	Not a heavy drinker^b^ (*n* = 445) % (95% CI)	Heavy drinker (*n* = 510) % (95% CI)	*P*‐value^c^
*Gender*				
Male	61.9 (58.9, 64.9)	42.8 (37.8, 48.0)	57.2 (52.0, 62.2)	**<0.01**
Female	38.1 (35.1, 41.1)	58.8 (49.9, 67.1)	41.2 (32.9, 50.1)	
*Age, years*				0.34
18–19	7.0 (5.2, 9.4)	53.6 (36.9, 69.5)	46.4 (60.5, 63.1)	
20–24	22.8 (18.8, 27.3)	50.7 (36.8, 64.5)	49.3 (35.5, 63.2)	
25–34	31.2 (28.6, 33.9)	40.2 (32.7, 48.3)	59.8 (51.8, 67.3)	
35–44	19.1 (17.0, 21.4)	51.2 (41.9, 60.4)	48.8 (39.6, 58.2)	
45–54	12.0 (10.1, 14.2)	57.4 (40.6, 72.6)	42.6 (27.4, 59.4)	
55–65	8.0 (5.8, 10.9)	45.3 (30.2, 61.3)	54.7 (38.7, 69.8)	
*Race/ethnicity*				**<0.01**
Black African	74.9 (67.9, 80.9)	43.9 (38.8, 49.1)	56.1 (50.9, 61.2)	
Coloured	4.0 (2.9, 5.5)	40.5 (29.1, 53.1)	59.5 (46.9, 70.9)	
White	19.6 (13.4, 27.8)	67.4 (52.8, 79.2)	32.6 (20.8, 47.2)	
Asian/Indian	1.4 (0.7, 2.7)	61.9 (27.3, 87.6)	38.1 (12.4, 72.7)	
*Urbanicity*				0.20
Urban	87.8 (80.4, 92.6)	49.3 (44.0, 54.7)	51.7 (45.4, 56.0)	
Rural	12.2 (7.4, 19.6)	42.8 (34.5, 51.6)	57.2 (48.4, 65.5)	
*Total annual personal income* d				0.88
Low	77.0 (73.6, 80.1)	47.7 (39.5, 56.1)	52.3 (43.9, 60.5)	
Medium	15.5 (12.9, 18.6)	44.4 (29.5, 60.3)	55.7 (39.7, 70.5)	
High	7.5 (5.4, 10.3)	49.9 (32.9, 66.9)	50.1 (33.1, 67.1)	
Total sample	—	48.4 (43.6, 53.2)	52.6 (46.8, 56.4)	—

Bold is used to indicate statistical significance

a Total is 1920 for the entire sample. Total sample size is 1487 for total personal income because 433 participants did not report income. Total sample size is 955 for heavy drinking because 965 participants did not report enough information to calculate heavy drinking.

b Heavy drinking defined as consuming ≥96 g (120ml) of absolute alcohol for men and ≥72 g (90 ml) of absolute alcohol for women at any location at least monthly.

c *P*‐value based on a corrected weight χ^2^ statistic transformed into an *F* statistic.

d Total annual personal income was categorised as low for R30 000 or less, medium as greater than R30 000 but less than or equal to R200 000 and high as greater than R200 000.

### Policy support

**Figure 1:**
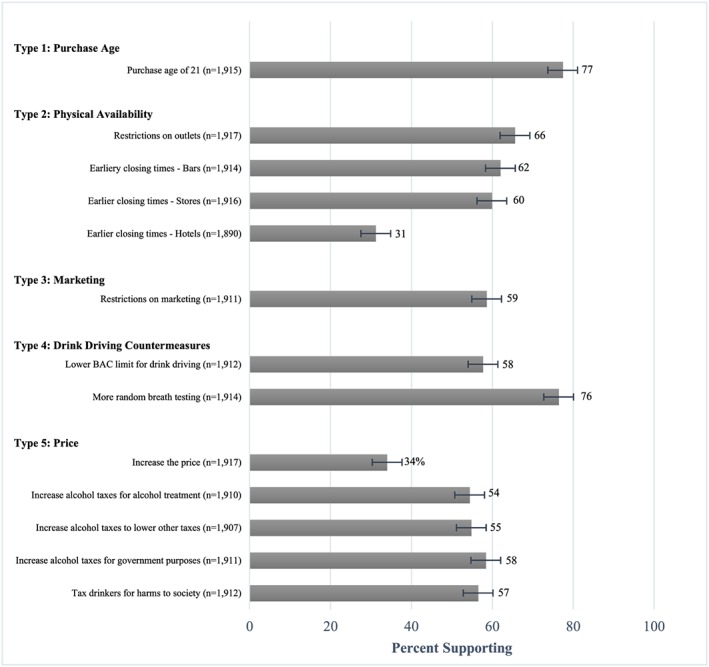
Support for alcohol policies. BAC, blood alcohol concentration. [Colour figure can be viewed at http://wileyonlinelibrary.com]

Support varied by alcohol policy, ranging from 31% to 77%, with support above 50% for 11 of the 13 policies (Figure 1). Support was higher for policies increasing the purchase age (77%), addressing drink driving (58–76%), restricting physical availability [60–66%, except for earlier closing times at hotels (31%)] and restricting marketing (59%). Support for the general item ‘increasing price’ was 34%, compared with 54–58% for the pricing policies for which more detail was given.

Compared with men, women were more likely to support increased use of random breath testing (RBT), increasing taxes for treatment, increasing taxes to lower other taxes (Table 2), policy support for increasing the minimum purchase age to 21 years, earlier bar closing times and restricting marketing varied by age. Persons aged 20–24 years (*P* = 0.005), 25–34 years (*P* < 0.001), 35–44 years (*P* < 0.001) and 45–54 years (*P* < 0.001) were more likely to support increasing the minimum purchase age than persons aged 18–19 years. Persons aged 45–54 years were more likely to support restricting marketing than persons aged 18–19 years (*P* = 0.011), and persons aged 25–34 years were more likely to support earlier bar closing times than persons aged 18–19 years (*P* = 0.008) and 20–24 years (*P* = 0.002). Policy support for increasing taxes to pay for any government services varied by income. Low‐income persons were more likely to support increasing taxes to pay for government services than were middle‐income persons (*P* < 0.001) or high‐income persons (*P* < 0.001). Heavy drinkers were less likely to support earlier liquor store closing and increasing the price of alcohol than were non‐heavy drinkers.

**Table 2:** Percent of participants supporting alcohol policies, by gender, heavy drinking status, income and age

Policy	Gender			Heavy drinking^b^			Total annual personal income^c^				Age (years)						
	Males (%)	Females (%)	*P*‐value^a^	Heavy drinker (%)	Not heavy drinker	*P*‐value	Low (%)	Medium (%)	High (%)	*P*‐value	18–19 (%)	20–24 (%)	25–34 (%)	35–44 (%)	45–54 (%)	55–65 (%)	*P*‐value
Increase purchase age to 21 years	75	81	0.07	51	49	0.72	77	16	7	0.75	5	21	33	20	13	7	**<0.001**
Restrict number of alcohol outlets	64	69	0.11	52	48	0.90	78	15	7	0.30	7	21	32	18	14	8	0.14
Earlier bar closing times	60	66	0.09	49	51	0.37	78	15	7	0.48	5	19	33	21	14	9	**0.02**
Earlier hotel closing times	30	33	0.18	56	44	0.10	81	11	8	0.14	7	23	29	19	14	8	0.72
Earlier store closing times	59	62	0.45	46	54	**0.02**	76	17	7	0.34	7	20	30	21	14	8	0.13
Restrict marketing	58	60	0.34	50	50	0.47	78	14	8	0.30	6	22	29	20	15	8	**0.02**
Lower blood alcohol concentration limit	58	57	0.61	53	47	0.24	75	17	8	0.22	7	21	33	18	12	9	0.51
More random breath testing	73	82	**0.02**	53	47	0.22	75	17	8	0.12	7	21	33	19	12	8	0.20
Increase the price	36	36	0.41	44	56	**0.03**	80	14	6	0.40	8	18	28	21	16	9	0.09
Increase taxes for treatment	52	59	**0.047**	48	52	0.12	79	15	6	0.36	8	22	32	18	14	6	0.31
Increase taxes to lower other taxes	52	60	**0.02**	51	49	0.84	77	16	7	0.60	7	23	32	17	14	8	0.29
Increase taxes for government purposes	56	62	0.08	49	51	0.40	84	12	5	**<0.001**	7	22	33	19	13	6	0.30
Tax drinkers to pay for harms	55	58	0.42	53	47	0.41	76	17	7	0.42	7	22	31	18	15	8	0.31

Bold is used to indicate statistical significance

a *P*‐value based on a corrected weight χ^2^ statistic transformed into an *F* statistic.

b Heavy drinking defined as consuming ≥120 ml of absolute alcohol for men and ≥90 ml of absolute alcohol for women at any location at least monthly.

c Total annual personal income was categorised as low for R30 000 or less, medium as greater than R30 000 but less than or equal to R200 000 and high as greater than R200 000.

## Discussion

More than 50% of adult drinkers indicated their support for 11 policy proposals. Support was highest for policies increasing the purchase age and addressing drink driving. Support was substantially lower for marketing restrictions and other pricing/tax policies, especially increasing price without specifying where the increase in taxes would be spent. The finding of less support for policies that would affect the economic availability of alcohol to drinkers has also been found from research conducted in the USA, Ireland and Australia with drinkers and non‐drinkers 12, 15, 18, 29, 30. The support for restrictions on alcohol advertisements was found to be lower in the South African sample than among persons in Norway and Australia 13, 15. This could be because the South African sample only included drinkers and also due to the high levels of lobbying in the media against a ban on alcohol advertising in 2012–2013.

Of the 13 alcohol control policies presented, there were three in which women were more likely than men to be supportive. Some age and income differences were also noted. These findings are consistent with previous studies in developed countries that found higher levels of support for certain alcohol policies among women 9, 11, 15, 16, 18, 19, older persons 9, 15, 18, 19 and less heavy drinkers 9, 11, 15, 16, 18. Like other studies, this research did not find that support for alcohol policies varied by income 9. We found no significant education effect in terms of policy support. International research findings have varied on this issue 11, 19.

The overall level of public support for alcohol policies appears higher in Tshwane than in recently collected estimates from South Koreans, 80% of whom were drinkers. The latter study found that 10 out of 12 policies had less than 50% support 22, the only exceptions being increasing the purchase age to 20 years (64% support) and increasing RBT to address drink driving (65%). Our findings, however, were in line with the high level of support for 15 alcohol policy interventions shown by younger and older adults (drinkers and non‐drinkers) in Cape Town, where support ranged from 60% for restrictions on alcohol marketing or advertising through sponsorships to 85% for earlier closing times for bars/taverns/shebeens and nightclubs 31. The level of policy support found in Tshwane was also in line with that reported in the earlier Puerto Rican study 23, that is, ranging from 67% to 95% across 20 items, and also levels reported in Brazil across six policy measures, 55% to 74% 24. A possible reason for the higher support afforded to a range of proposed alcohol policies in South Africa compared with South Korea could be the greater sense of a need for action given the very high burden associated with alcohol‐related harm in South Africa 2 and the high levels of media attention given to alcohol policy reform in recent years.

Across the five studies conducted in developing countries, there appears to be substantial support for raising the purchase age of alcohol, ranging from 55% in Brazil 24 to 84% in Puerto Rico 23, and for more RBT of drivers, ranging from 65% in South Korea 22 to 83% in the Cape Town study 31. Support for increasing prices varied more across countries (e.g. 75% in the Cape Town study and only 21% in South Korea). In most of the countries, there was substantial support for increasing restrictions on marketing (e.g. 58% in the Tshwane study and 60–65% in the Cape Town study), 74% to 82% in Puerto Rico and 55% to 68% in Brazil. In contrast, only 41% of participants in South Korea indicated that they supported restrictions on marketing. It should be noted that the South Korean, Brazilian and Puerto Rican studies also included non‐drinkers, so the higher level of support for various policy measures in these countries is to be expected given the finding that drinking status is inversely associated with support for alcohol control measures 9, 11, 15, 16, 18.

Drinkers in Tshwane, nonetheless, support many of the controls on alcohol that are being considered in South Africa, for example, increasing the purchase age to 21 years (77%), implementing greater restrictions on liquor outlets (66%), increasing restrictions on marketing of alcohol (58%) and lowering blood alcohol concentration levels for drivers (58%). In line with prior research 9, 15, 16, the data suggest that when people might be more directly (negatively) affected by a given policy measure their support for that policy is likely to be less. For example, 18‐ to 20‐year‐olds were less supportive of raising the minimum purchase age than drinkers aged 25–34 years, were less supportive of earlier bar closing times than older persons and were less supportive of increasing alcohol taxes for government purposes than 35‐ to 54‐year‐olds. Similarly, heavy drinkers were less likely to support earlier liquor store closing and increasing the price of alcohol.

This study is subject to various limitations. First, the data are specific to the Tshwane Metropole, and it is unknown whether they generalise to other parts of South Africa, particularly rural areas. However, given the synergy with the findings of Ferrell's research in Cape Town 30, it does seem as if the findings may generalise to other metropolitan areas at least. Future research needs to assess the views of populations in deeper rural areas and also of non‐drinkers whose views should also be given consideration given the high proportion of non‐drinkers and the growing awareness of the harm caused by drinkers to non‐drinkers 32. This study also uses cross‐sectional data, so the analysis is unable to determine the direction of causality 12.

Furthermore, the question on restricting alcohol advertising restrictions was not specific. Therefore, it is unknown whether participants are in favour of a total alcohol advertising ban that is currently under consideration 8, although they are clearly mostly in favour of further restrictions on advertising. The same criticism regarding specificity could be levelled at the question about ‘restricting outlets’. Having the study participants respond to questions asked by interviewers could also have introduced a desirability bias, but the very high levels of heavy drinking reported suggests that this was not the case. As indicated in the Methods, there was no complete consumption and income data on all drinkers who were sampled. This may mean that the findings for consumption and income might have differed had data on consumption in all locations and for all income groups been equally available for all subgroups.

## Conclusion

These findings demonstrate that policymakers need not fear public backlash from all drinkers when increasing controls on alcohol. Some resistance is likely from those drinkers more directly affected by particular policies. Given the importance of alcohol policy reform in South Africa to bring down consumption and related harms, policymakers should use public support for reforms where they exist, but not be deterred when resistance comes for particular polices from those likely to be more affected.

## Conflict of Interest

The authors have no conflicts of interest.
